# A study on green synthesis, characterization of chromium oxide nanoparticles and their enzyme inhibitory potential

**DOI:** 10.3389/fphar.2022.1008182

**Published:** 2022-10-07

**Authors:** Zainab, Shujaat Ahmad, Idrees Khan, Khalid Saeed, Hanif Ahmad, Aftab Alam, Mazen Almehmadi, Ahad Amer Alsaiari, Yu Haitao, Manzoor Ahmad

**Affiliations:** ^1^ Department of Chemistry, University of Malakand, Charsadda, Khyber Pakhtunkhwa, Paksitan; ^2^ College of Chemistry and Materials Science, Hebei Normal University, Shijiazhuang, China; ^3^ Department of Pharmacy, Shaheed Benazir Bhutto University Sheringal, Dir (Upper), Khyber Pakhtunkhwa, Pakistan; ^4^ Department of Chemistry, Bacha Khan University, Charsadda, Khyber Pakhtunkhwa, Pakistan; ^5^ Department of Clinical Laboratory Sciences, College of Applied Medical Sciences, Taif University, Taif, Saudi Arabia

**Keywords:** chromium oxide nanoparticles, green synthesis, enzyme inhibition, characterization, cholinesterase inhibition

## Abstract

The conventional chemical methods of nanoparticles synthesis have been effectively replaced by nanoparticle synthesis mediated by plants. The current study describes the environmental friendly synthesis of chromium oxide nanoparticles (Cr_2_O_3_ NPs) using *Erythrophleum guineense* plant extract. The synthesis of Cr_2_O_3_ NPs was validated by UV/VIS spectroscopy, Energy Dispersive X-Ray (EDX), Scanning Electron Microscopy (SEM), and X-ray diffraction (XRD) studies. The appearance of the Sharpe peak at 460 nm in the UV/Vis spectrum and the colour change caused by surface plasma resonance confirmed the formation of Cr_2_O_3_ NPs. The EDX spectrum of Cr_2_O_3_ nanoparticles revealed the presence of carbon, oxygen, and chromium, while SEM analysis revealed an irregular round morphology (with a size below 400 nm). In addition, XRD studies suggested their crystalline nature by the characteristic peaks at 34° and 36° and 42° (2Ɵ), respectively. The green synthesized Cr_2_O_3_ NPs showed promise as *in-vitro* cholinesterase inhibitor at tested concentrations (62.5–1,000 μg/ml), with IC_50_ values of 120 and 100 μg/ml against Acetylcholinesterase (AChE) and Butyrylcholinesterase (BChE), respectively. The results suggested that the green synthesized Cr_2_O_3_ NPs could be used in the future to stop enzyme from working and for other biological activities.

## Introduction

Nanotechnology is an emerging field in biomedical sciences as the number of its applications in health care sciences increases (Khan et al., 2016a). Nanotechnology is a fast expanding field that utilizing nanomaterials for diagnosis and treatment purposes (Sargazi et al., 2022). The development of nanomaterials is one of the most promising advances for the treatment of a wide range of illnesses, such as fungal and bacterial infections, as well as various types of cancer (Khan et al., 2016b; Khan et al., 2017b). Nanoparticles (NPs) remarkable physicochemical features, including minute sizes, large surface-to-volume ratios, and size-dependent optical properties, have received considerable attention in potential increased biological applications (Sengul and Asmatulu, 2020; Yaqoob et al., 2020). NPs are the building blocks of nanotechnology and nanomedicine, they have been employed in a variety of applications, including diagnosis, detection, drug delivery, and treatment of various diseases, and treatment of different types of cancer (Khan et al., 2017a). For example, metal oxide NPs are utilized extensively in various products for different purposes (Khan et al., 2015), like photocatalysis (Mahmood et al., 2021) adsorption (Riaz et al., 2022), and enzyme inhibition (Ashraf et al., 2021) etc.

Due to their excellent stability, hardness, high resistivity, high melting temperature, and wide bandgap of 3.4 eV, chromium oxide nanoparticles (Cr_2_O_3_) have grained particular among metal oxide NPs (Iqbal et al., 2020; Isacfranklin et al., 2020). Cr_2_O_3_ NPs could be used in materials for catalysis (Mori et al., 2017), photocatalysis (Zelekew et al., 2021), super capacitors (Shafi et al., 2021), lithium ion batteries (Cao and Zuo, 2017), sensing (Gao et al., 2019), and other biological activities due to their unique properties (Talat et al., 2021). Biocompatibility of Cr_2_O_3_ nanoparticles is an essential parameter for their use in many biological systems (Khan et al., 2021). Medically Cr_2_O_3_ NPs were significantly utilized as potent anti-oxidants, anti-bacterial, anti-cancer, anti-viral and anti-diabetic etc. (Rayani Nivethitha and Carolin Jeniba Rachel, 2020; Ghotekar et al., 2021; Khan et al., 2021), drug delivery (Azizi, 2020) etc. Due to the substantial usage of hazardous chemicals, both as solvents and starting materials, along with considerable heat and pH fluctuations, the Cr_2_O_3_ NPs prepared by conventional chemical or physical procedures exhibits a number of disadvantages (Bahrulolum et al., 2021; Zainab et al., 2021). The usage of such parameters imparts additional hazardous properties, including as carcinogenicity and environmental toxicity, that restrict the deployment of nanoparticles in various clinical and biomedical applications (Zhang et al., 2020).

Thus the green synthesis of nanoparticles has developed as an alternative to conventional physical and chemical methods and has the potential to mitigate some of their damaging impacts (Ahmad et al., 2022). Green synthesis is a fascinating method for producing nanoparticles since it is straightforward, cost-effective, and eco-friendly (Aravind et al., 2021). Biological synthesized metallic NPs are cancer-fighting cytotoxic agents (Patil and Chandrasekaran, 2020). Compared to the synthesis of nanoparticles by bacteria and fungus, the synthesis of nanoparticles using plant extracts is a simple and straightforward approach for producing nanoparticles on a large scale (Singh et al., 2018). Numerious researchers are now interested in green synthesis of chromium oxide nanoparticles (Cr_2_O_3_ NPs) using plant extracts (Liu et al., 2021). *Callistemon viminalis* (Bottle Brush), flower extracts (Hassan et al., 2019), *Tridax procumbens* Leaf Extract (Ramesh et al., 2012), cactus (*Opuntia ficus-indica*) plant extract (Tsegay et al., 2021), etc. have been used in the production of Cr_2_O_3_.

The widely distributed plant *E. guineense* belongs to the Caesalpiniaceae family and has been used to treat cardiovascular diseases owing to its analgesic and anti-inflammatory characteristics (Ngounou et al., 2005). *E. guineense*, which is phytochemically abundant in glycosidic alkaloids and polyphenols (Nwude and Chineme, 1980; Adeoye and Oyedapo, 2004) is an effective agent for reducing metal ions. Prior to this study, various plant extracts were used for the green synthesis of Cr_2_O_3_ NPs, including *Hyphaene thebaica* (Mohamed et al., 2020), *Rhamnus virgata* (J et al., 2020), *Cannabis sativa* (Sharma and Sharma, 2021), *Manihot esculenta* (Thara et al., 2022) but there was no report on the use of *E. guineense* extract. In the present studies, *E. guineense* extract mediated Cr_2_O_3_ NPs were synthesized and characterized by UV/Vis spectroscopy, EDX, SEM, and XRD techniques along with evaluation of them *in vitro* cholinesterase inhibitory potential.

## Experimental work

### Materials

As chemical, methanol (commercial grade, double distilled), deionized water, and Chromium chloride (Sigma, Germany) were used as chemicals**.**

### Samples collections and preparation

Professor Dr. Mehboob Ur Rehman, Department of Botany, Jehanzeb Post Graduate Collage, Swat, KPK, Pakistan, gathered 4 km of *E. guineense* aerial parts from his natural habitat in Nigeria after carefully identifying them. A voucher specimen (accession # EG016o the department’s herbarium.

The materials were ground to powder using an electrical blender and extracted thrice with 80% methanol in a cold maceration manner (3 × 10 days). The crude extract was obtained by concentrating on a rotary evaporator (40°C) and was used to synthesize Cr_2_O_3_ NPs.

### Synthesis of chromium nanoparticles

A crude extract solution of 0.5% *E. guineense* was prepared in deionized water and filtered thrice to remove any suspended particles. The clear solution of plant extract and chromium chloride salt solution (0.2 M) were placed in a 250 ml conical flask (ratio of 3:7) and were magnetically stirred for 1.5 h at 100°C. The formation of NPs was thoroughly monitored by color change. After allowing the solution to cool, the Cr_2_O_3_ NPs were separated by centrifugation (13,500 rpm at 4°C) followed by drying in an oven (80°C). The maximum nanoparticles formed in a suspension was determined using UV-Visible spectrophotometer.

### Characterization

The UV/VIS study of Cr_2_O_3_ NPs was obtained through a UV/Vis spectrophotometer (UV-1800, Shimadzo, Japan), while the EDX analyses were carried out on an EDX machine (INCA 200, Oxford instruments, UK). The surface morphology of Cr_2_O_3_ NPs was performed on SEM [JEOL, JSM-5910 SEM (high vacuum mode)]. The phase and crystal structure analysis were performed through XRD (JDX-3532, JEOL, Japan).

### Acetylcholinesterase and butyrylcholinesterase inhibition assays

Sigma Aldrich provided all of the enzymes and chemicals required for the experiment. The enzyme (AChE/BChE) inhibition activities were measured using the spectrophotometric method as described in the literature [21]. Throughout, the reported protocol and assay conditions were followed [22]. This experiment included several dilutions of Cr_2_O_3_ NPs (1,000, 500, 250, 125, and 62.5 μg/ml). The substrates butyryl choline chloride (BChCl) and acetyl choline iodide (AChI) were used to evaluate BChE and AChE inhibitory activities, respectively.

Briefly, solution A containing DTNB (0.2 mM) in 62 mM sodium phosphate buffer (pH 8.0, 880 μl) was combined with solution B, the test sample (40 μl), and either acetyl cholinesterase or butyryl cholinesterase solution (40 μl) and incubated at 25°C 15 min. The experimental reactions were initiated by the addition of 40 μl ACh or BCh, and the hydrolysis of ACh or BCh was witnessed at 412 nm with (BMS-USA) spectrophotometer. Concentrations of the tested compounds which inhibit the hydrolysis of ACh and BCh (substrates) by 50% (IC_50_) were determined by observing the effect of an increase in concentrations of the compound on the inhibition values (Ellman et al., 1961; Rocha et al., 1993).

## Results and discussion

### UV/Vis study

**FIGURE 1:**
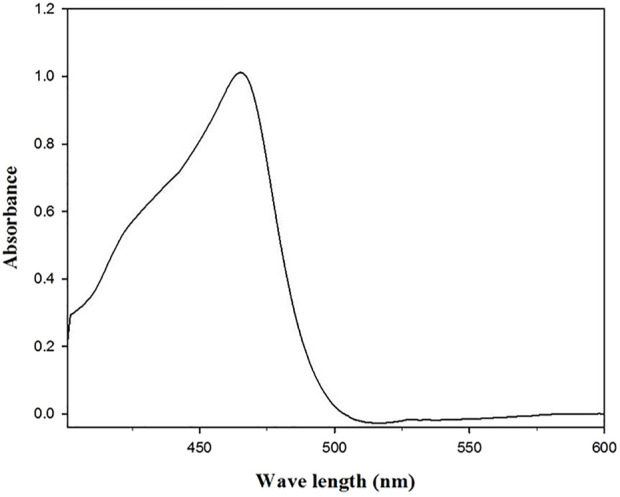
UV-Vis spectra of the green synthesized chromium nanoparticles.

Also, UV/Vis spectrophotometry was used to confirm the synthesis of chromium oxide nanoparticles. Figure 1 depicts the UV/Vis spectra of chromium oxide nanoparticles extracted from *E. guineense*. In the visible spectrum, there was a prominent peak at around 460. The emergence of peaks indicates that plant extract greatly decreased the quantity of chromium salt into oxide nanoparticles. Similarly, Jaswal et al. (2014) also reported a similar UV/Vis spectrum for chromium oxide nanoparticles synthesized by the precipitation method from their precursor.

### Elemental composition analysis

**FIGURE 2:**
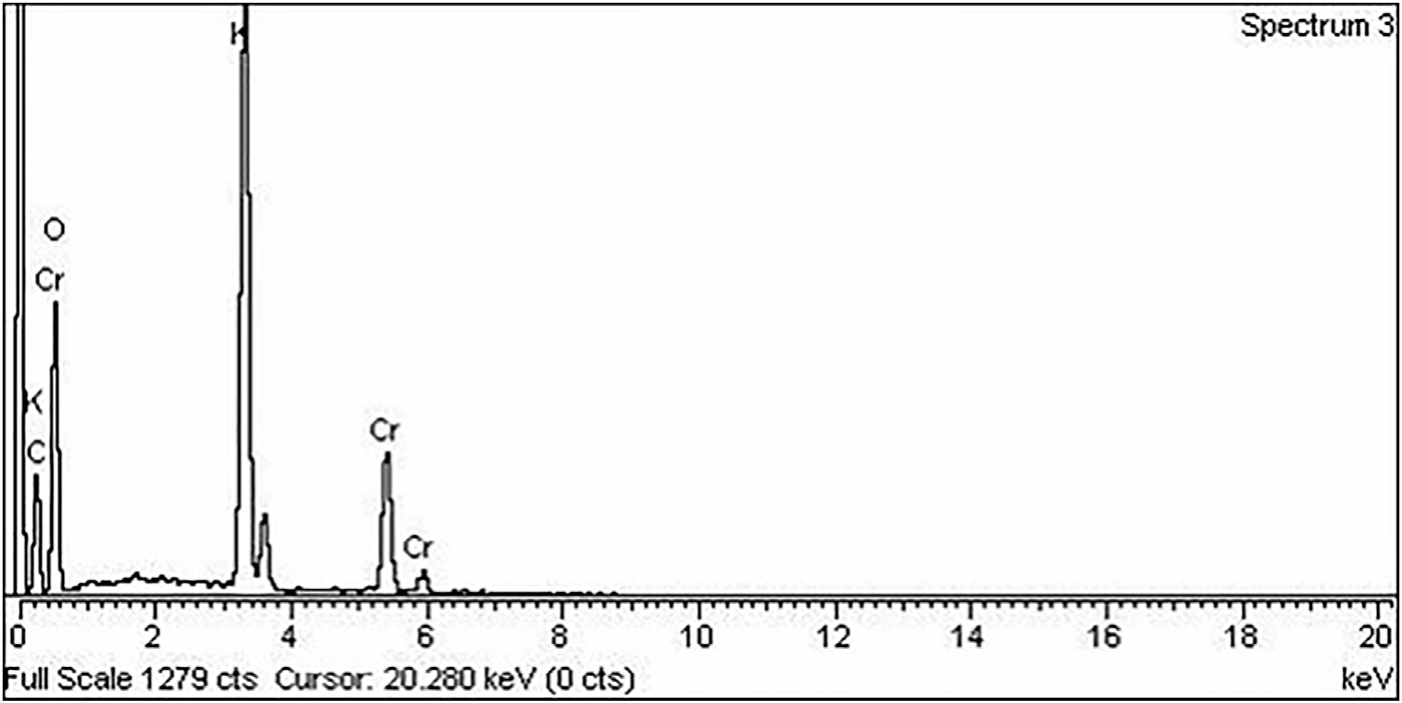
EDX spectra of the green synthesized chromium nanoparticles.

EDX is a type of analysis that is used to determine which elements are in a compound or material. Figure 2 depicts the EDX spectrum of green synthesized chromium nanoparticles and shows the presence of carbon, oxygen, and chromium in our synthesized Cr_2_O_3_ NPs.

### Surface morphology studies

**FIGURE 3:**
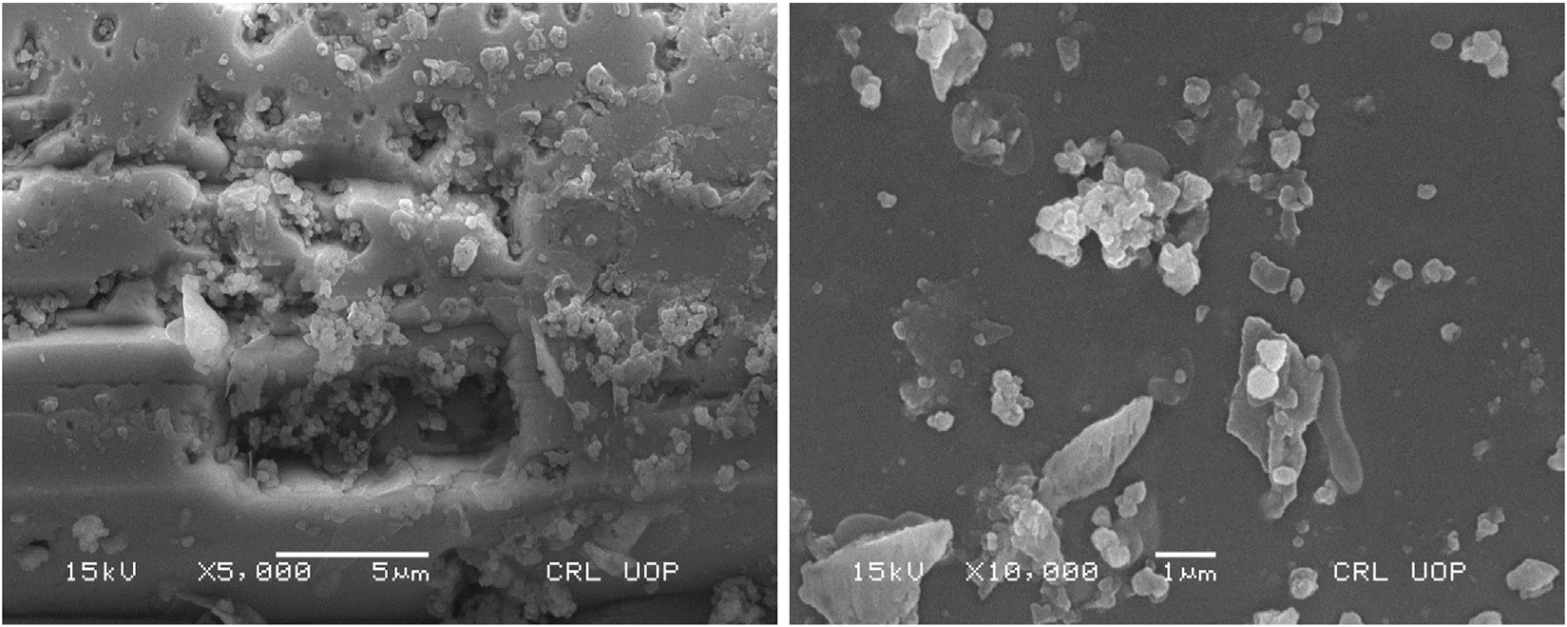
SEM images of the green synthesized chromium oxide nanoparticles.

The SEM data is useful in obtaining information about nanascale materials, especially the shapes, sizes, dispersion, and other surface phenomena. The SEM also gives useful information regarding surface contaminations, spherulites, lamellae, crystallinity, and qualitative chemical analyses. Figure 3 shows the SEM micrographs of chromium nanoparticles. The SEM images depicts that the Cr_2_O_3_ NPs formed irregular round-shaped particles and dispersed as aggregates in sizes below 400 nm.

### XRD study

**FIGURE 4:**
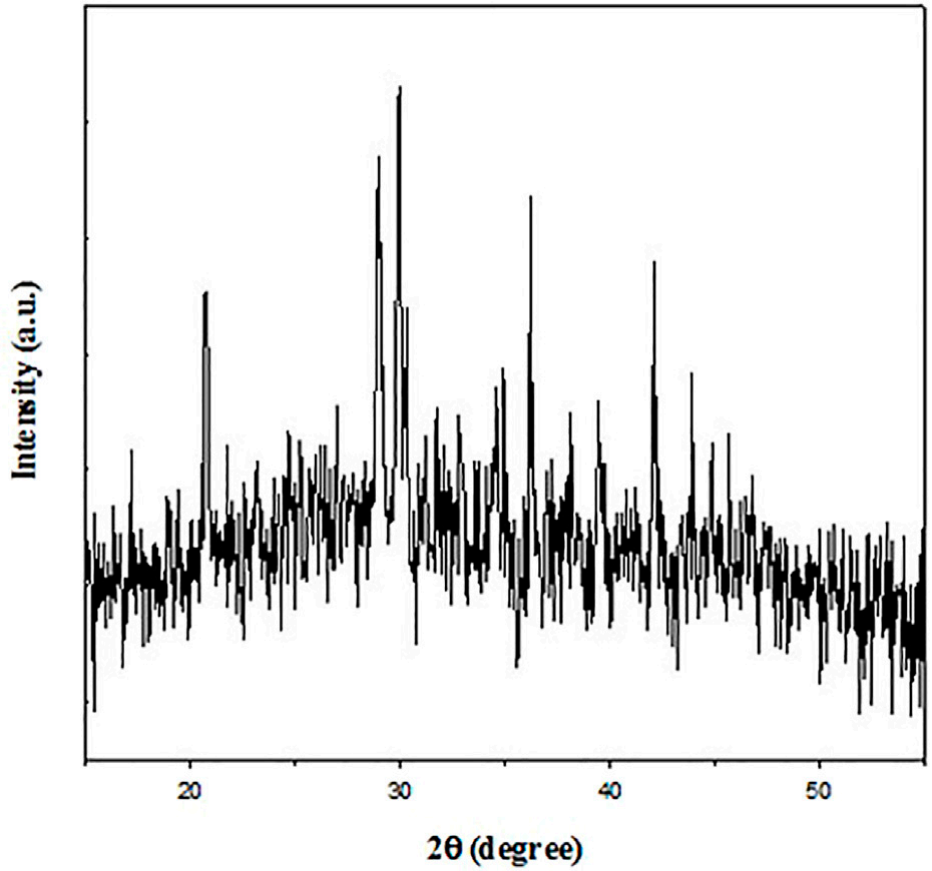
XRD spectrum of the green synthesized chromium oxide nanoparticles.

XRD is a useful tool for characterizing nanomaterials and determining sample size and shape; identifying crystalline phases; preferential order and epitaxial growth of crystallites; and spacing between lattice planes. The XRD technique also able to differentiate crystalline, semi-crystalline, and amorphous materials. The XRD study was performed to determine the crystalline nature of Cr_2_O_3_ NPs. The XRD spectrum (Figure 4) presented characteristic peaks at 34°, 36°, and 42° (2Ɵ values), thus confirming the crystalline nature of our synthesized Cr_2_O_3_ NPs (Tsuzuki and McCormick, 2000).

### Anticholinesterase inhibition of Cr_2_O_3_ nanoparticles

The neurotransmitters play a central role in the coordination of the body. According to the neuronal classification on the basis of neurotransmitters, the cholinergic system has a major role in the cognitive functions of the brain (Schliebs and Arendt, 2011). The degeneration of neurons or the low secretion of acetylcholine in the brain, particularly in the hippocampus, leads to cognitive disorders. Alzheimer’s disease is one of the leading cognitive dysfunctions (Parri et al., 2011). The anticholinesterase compounds have been proven to be effective in the mitigation of the symptoms of Alzheimer’s disease. These compounds block the enzyme responsible for the breakdown of acetylcholine and so increase their quantity and avoid the associated symptoms (Gadzhanova et al., 2010). A variety of bioactive compounds have been explored for their anticholinesterase potential. Wide attention has been focused on the synthesis and isolation of bioactive compounds having high efficacy against Alzheimer’s disease (Zeb et al., 2014; Ahmad et al., 2015; Sadiq et al., 2015). The solubility and bioavailability of the drug play an important role in the enhancement of the efficacy of the drug. The bioavailability in turn depends upon the particle size of the drugs used (Judy et al., 2012).

Table 1 represents the enzyme inhibitory potential of green synthesized Cr_2_O_3_ NPs. The Cr_2_O_3_ NPs in these experiments have shown good acetylcholinesterase (AChE) and butyrylcholinesterase (BChE) inhibitory activities. The highest activity recorded for these NPs against AChE was 71% at 1,000 μ/ml, followed by 64, 57, 51, and 43% at 500, 250, 125, and 62.5 g/ml, respectively. IC_50_ values shown by the test samples is 120 μ/ml while for the positive control galantamine IC_50_ was recorded as 17 μg/ml. These activities are almost similar to previously reported results for Bottlebrush flowers extract as a reducing agent (Hassan et al., 2019). These NPs also inhibited BChE at the highest concentration used; 73% of the enzyme was inhibited, while at the lowest concentration, 45% of the enzyme was inhibited. IC_50_ shown by the test NPs is 100 μg/mL as compared to the standard (18 μg/ml), lower than the previously reported (132.24 μg/ml) for plant synthesized NPs of chromium oxide (Hassan et al., 2019).

**TABLE 1:** Anti-cholinesterase inhibitory potential of chromium NPs compared with galantamine used as a standard.

Compound name	Concentration μg/ml	% AChE inhibition	IC_50_ μg/ml	% BChE inhibition	IC_50_ μg/ml
**Chromium NPs**	1,000	71	120	73	100
	500	64		65	
	250	57		59	
	125	51		53	
	62.5	43		45	
**Galantamine**	1,000	81	17	85	18
	500	75		79	
	250	71		71	
	125	65		67	
	62.5	60		62	

## Conclusion

Green synthesis was used to produce Cr_2_O_3_ NPs from their precursors, with *E. guineense* plant extracts serving as reducing agents. The morphological study illustrated that the size (below 400 nm) of chromium nanoparticles was similar while the *E. guineense* mediated chromium oxide nanoparticles were irregular and round in shapes. The EDX spectra represents peaks for carbon and oxygen along with chromium, thus confirming the reduction of chromium from precursor slat. The XRD and UV-VIS studies further confirmed the green synthesis of chromium oxide nanoparticles. The promising AChE and BChE inhibitory capabilities of our synthesised chromium oxide nanoparticles implies that such NPs could be used therapeutically in neurodegenerative illnesses, including Alzheimer’s disease.

## Data Availability

The original contributions presented in the study are included in the article/supplementary material, further inquiries can be directed to the corresponding authors.
